# Design and evaluation of a unique SYBR Green real-time RT-PCR assay for quantification of five major cytokines in cattle, sheep and goats

**DOI:** 10.1186/s12917-015-0382-0

**Published:** 2015-03-17

**Authors:** Carinne Puech, Laurence Dedieu, Isabelle Chantal, Valérie Rodrigues

**Affiliations:** INRA, UMR1309 CMAEE, Montpellier, F-34398 France; CIRAD, DGD-RS-Dist, Montpellier, F-34398 France; CIRAD, UMR Intertryp, Montpellier, F-34398 France; CIRAD, UMR CMAEE, Montpellier, F-34398 France

**Keywords:** Cytokine expression, SYBR Green RT-qPCR, Cattle, Sheep, Goats, Immune response, Th1/Th2 response, Reference genes

## Abstract

**Background:**

Today, when more than 60% of animal diseases are zoonotic, understanding their origin and development and identifying protective immune responses in ruminants are major challenges. Robust, efficient and cost-effective tools are preconditions to solve these challenges. Cytokines play a key role in the main mechanisms by which the immune system is balanced in response to infectious pathogens. The cytokine balance has thus become the focus of research to characterize immune response in ruminants. Currently, SYBR Green reverse transcriptase quantitative PCR (RT-qPCR) is the most widely method used to investigate cytokine gene expression in ruminants, but the conditions in which the many assays are carried out vary considerably and need to be properly evaluated. Accordingly, the quantification of gene expression by RT-qPCR requires normalization by multiple reference genes. The objective of the present study was thus to develop an RT-qPCR assay to simultaneously quantify the expression of several cytokines and reference genes in three ruminant species. In this paper, we detail each stage of the experimental protocol, check validation parameters and report assay performances, following MIQE guidelines.

**Results:**

Ten novel primer sets were designed to quantify five cytokine genes (IL-4, IL-10, IL-12B, IFN-γ and TNF-α) and five reference genes (ACTB, GAPDH, H3F3A, PPIA and YWHAZ) in cattle, sheep, and goats. All the primer sets were designed to span exon*-*exon boundaries and use the same hybridization temperature. Each stage of the RT-qPCR method was detailed; their specificity and efficiency checked, proved and are reported here, demonstrating the reproducibility of our method, which is capable of detecting low levels of cytokine mRNA up to one copy whatever the species. Finally, we checked the stability of candidate reference gene expression, performed absolute quantification of cytokine and reference gene mRNA in whole blood samples and relative expression of cytokine mRNA in stimulated PBMC samples.

**Conclusions:**

We have developed a novel RT-qPCR assay for the simultaneous relative quantification of five major cytokines in cattle, sheep and goats, and their accurate normalization by five reference genes. This accurate and easily reproducible tool can be used to investigate ruminant immune responses and is widely accessible to the veterinary research community.

**Electronic supplementary material:**

The online version of this article (doi:10.1186/s12917-015-0382-0) contains supplementary material, which is available to authorized users.

## Background

Animal diseases have major economic, sanitary, health and environmental consequences worldwide. In 2013, the World Organization for Animal Health (OIE) calculated that more than 60% of animal diseases are zoonotic and that 75% of them are responsible for human emerging infections [1]. Infectious diseases are at the animal-human-ecosystem interface and are a major public health concern, particularly emerging infectious diseases [2]. The importance of ruminant livestock in sustainable agricultural systems [3] and the impact of major livestock diseases on food production is no longer the subject of debate [4]. However, developing efficient control strategies is a major challenge, and understanding disease pathogenesis and identifying protective immune responses are prerequisites, requiring robust, efficient, and cost-effective tools.

In response to pathogens, the immune system is regulated by complex mechanisms in which cytokines play a key role. They control the activation, proliferation and/or differentiation of different types of cells as well as in the secretion of antibodies and mediators [5]. Differential cytokine production and expression, in particular the Th1/Th2 balance, has thus become a widely used way to characterize disease pathogenesis in cattle [6,7], goats [8] and sheep [9,10].

The cytokine balance is routinely characterized either by quantification of secreted cytokine proteins or by the expression of cytokine messenger RNA (mRNA). A few optimized assays are currently available for the measurement of bovine and ovine cytokines secreted: ELISA kits, monoclonal antibodies and standards have been developed and marketed including interferon-gamma, (IFN-γ), tumor necrosis factor-alpha (TNF-α) [11], interleukin (IL)-10 [12], IL-12 [13] and IL-4 [14]. For the measurement of caprine cytokines secreted, most of the few specific tools available are based on cross-reactivity with other ruminants, but are not always sure [15]. Using the ELISA method for ruminants is costly and only a limited number of cytokines can be analyzed from a single sample. In addition, despite technological advances, veterinary immunologists working on ruminants lack tools to investigate host immunity [15-17]. Cost-effective tools able to quantify several cytokines in several species of ruminants would be a significant advance.

To achieve this objective, analyzing cytokine gene expression has become a widely used method to establish a cytokine profile in ruminants. Currently, reverse transcriptase quantitative polymerase chain reaction (RT-qPCR) is the routine method used to investigate gene expression. RT-qPCR makes it possible to simultaneously characterize different genes, using small quantities of sample with high specificity, sensitivity and accuracy [18,19]. Beside the specific probe strategy, the use of SYBR Green dye is also a sensitive, robust and reproducible method, provided that primer design and set up conditions are properly optimized. In addition, the SYBR Green method is cheaper than methods using probes, and is thus more widely and easily transferable. However, the wide range of published studies on ruminants cites many different procedures and conditions. Most of these assays are specific to only one species and some were even published without reporting all validation parameters.

The objective of our study was thus to develop an innovative and cost-effective RT-qPCR assay, to simultaneously quantify the expression of five major cytokines involved in Th1/Th2 responses, IL-4, IL-10, IL-12B, IFN-γ and TNF-α, using a single primer set and hybridization temperature for three species of ruminants, cattle, sheep and goats, in a single assay. Here we detail each stage of the experimental protocol used for the development of our SYBR Green RT-qPCR assay and offer a reliable and transferable tool. These stages range from template preparation, nucleic acid quality, reverse transcription (RT) strategy up to the PCR conditions and data reporting [20], and checking validation parameters and assay performances following the recommendations of the Minimum Information for publication of Quantitative real-time PCR Experiments (MIQE) guidelines [21]. We carefully selected five reference genes for accurate quantification of gene expression by RT-qPCR and checked the stability of their expression. The identification of internal controls to normalize gene expression is essential for relative gene expression [22], and normalization by multiple reference genes instead of one is recommended [23]. For that reason, we chose the reference genes most commonly used and/or described as stable in different types of samples. *Glyceraldehyde 3-phosphate dehydrogenase* (GAPDH) and *beta*-*actin* (ACTB) are the most commonly used reference genes. These genes have been described as optimal reference genes in bovine peripheral blood mononuclear cells (PBMC) [24] or whole blood [25] and thus can be used to normalize gene expression. Previous studies also showed that *Tyrosine 3-monooxygenase/tryptophan 5-monooxygenase activation protein, zeta polypeptide* (YWAHZ) [26,27], *Peptidylprolyl Isomerase A* (PPIA) [26,28] or *H3-histone family 3A* (H3F3A) [29] are the most stable genes in bovine peripheral lymphocytes and ovine whole blood.

The SYBR Green RT-qPCR assay we developed can be used for cattle, sheep and goats to quantify the expression of five major cytokine genes (IL-4, IL-10, IL-12B, IFN-γ and TNF-α) and five candidate reference genes (GAPDH, ACTB, YWHAZ, PPIA and H3F3A). Our SYBR Green RT-qPCR assay is based on an optimized and validated protocol and is an easily reproducible and reliable tool specifically designed to investigate immune response in ruminants.

## Methods

In this paper, we use the nomenclature proposed by the Minimum Information for publication of quantitative real-time PCR experiments (MIQE) guidelines [19]. Candidate genes used for normalization are referred to as reference genes and the fractional PCR cycle used for quantification was Cq to quantification cycle.

### Animals, blood collection and ethical considerations

Samples from healthy crossbreed cattle, Suffolk sheep and Saanen goats (three animals per species) were collected in an animal housing facility (Montpellier–France). Whole blood was collected from the jugular vein either with Tempus™ Blood RNA tubes (Applied Biosystems Ltd., Warrington, UK) or heparinized BD Vacutainer® tubes (Beckton Dickinson, New Jersey, USA) according to the manufacturer’s instructions. Experimental procedures for animal maintenance and blood sampling were approved by the Languedoc-Roussillon regional ethics committee (French CE-LR #36) in the Authorised Project using animals for scientific purposes #12ANI01.

### Tempus™ whole blood total RNA samples

Total RNA was extracted from whole blood of cattle, sheep and goats with Tempus™ Blood RNA System (Applied Biosystems®, Warrington, UK). After blood collection, RNA was immediately extracted using the Tempus™ Spin RNA Isolation Reagent Kit according to the manufacturer’s instructions. All the samples were treated with DNAse, AbsoluteRNA Wash Solution, as recommended in the kit. RNA samples were stored at –80°C until conversion into cDNA. These samples are henceforth referred to as « Tempus™ total RNA ».

### Isolation, stimulation and RNA extraction of PBMC

Peripheral blood mononuclear cells (PBMC) were isolated from heparinized whole blood by density gradient centrifugation. Briefly, PBMCs were collected after centrifugation on Histopaque®-1077 (Sigma Aldrich, France) for sheep and goats and on Histopaque®-1083 (Sigma Aldrich, France) for cattle, according to the manufacturer’s instructions. Cell viability was assessed by Trypan blue exclusion. PBMCs were resuspended in RPMI 1640 Medium-glutaMAX™ (Life Technologies™, USA) supplemented with 5.10^−5^ M βmercaptoethanol (Life Technologies™, USA), 50 μg/ml gentamycin (Life Technologies™, USA) and 10% heat inactivated fetal calf serum (FCS; Eurobio AbCys, France) and were seeded in 12-well plates at 2.10^6^ cells/ml. PBMCs were cultured in medium (unstimulated condition) or stimulated with 5 μg/ml of Concanavalin A (stimulated condition) for 36 h at 37°C and 5% CO_2_. Total RNA was extracted using the RNeasy® Mini Kit (Qiagen Ltd., Crawley, UK) and treated with RNase-free DNase Set (Qiagen Ltd., Crawley, UK) for 30 min at room temperature. RNA samples were stored at –80°C until conversion into cDNA.

### RNA quantification and quality control

The purity and quantity of RNA were assessed using a NanoDrop™ ND-1000 Spectrophotometer (Thermo Fisher Scientific, MA, USA). The A260:A280 ratio ranged from 2.1 to 2.2. RNA integrity was checked with the Agilent 2100 Bioanalyzer using the RNA 6000 Nano Assay Kit (Agilent Technologies, Inc. Santa Clara, USA). The RNA Integrity Number (RIN) ranged from 7 to 9.6.

### Reverse transcription reaction

Two hundred nanograms of total RNA from each sample were reverse transcribed with the AffinityScript QPCR cDNA synthesis kit (Agilent Technologies, Inc. Santa Clara, USA) using oligo-dT strategy, and according to the manufacturer’s instructions. cDNA samples were stored as multiple aliquots at –20°C for subsequent use. No-reverse transcription (no-RT) controls (assay without reverse transcriptase) were also prepared to check for non-specific amplification.

### Cytokine and gene reference primers set design

All oligonucleotides were synthesized by Eurogentec (Seraing, Belgium).

Primers were designed by hand to span exon*-*exon boundaries and to fulfill the following criteria: located near the 3′ end, GC% between 40 and 70%, giving an approximately 200 bp amplicon and a melting temperature between 62 and 65°C. Exon spanning primers were designed by aligning *Bos taurus* gene sequences from National Center for Biotechnology Information (NCBI) GenBank database (btau 4.6.1) with mRNA-to-genomic alignment program, Spidey (www.ncbi.nlm.nih.gov/spidey). Species homologies and exon positions of ovine and caprine primers were checked *in silico*, respectively, with *Capra hircus* (chir_1.0) and *Ovis aries* (oar_V3.1) gene sequences from NCBI GenBank. The absence of primer-dimers and secondary structures was checked *in silico* using the OligoCalculator, an online oligonucleotide sequence calculator (Sigma Aldrich Co., St. Louis, USA).

Accession numbers and the exon location of each species are listed in Table 1 and primer sequences in Table 2.

**Table 1:** **Accession number and exon locations of primers for bovine, caprine and ovine gene expression**

**Target gene**	**Species**	**Accession number** ^**a**^	**Exon position**	
			**Primer F**	**Primer R**
**IL-4**	*Bos taurus*	NM_173921	E2	E3-E4
	*Capra hircus*	NM_001285681	E2	E3-E4
	*Ovis aries*	NM_001009313	E2	E3-E4
**IL-10**	*Bos taurus*	NM_174088	E2-E3	E5
	*Capra hircus*	XM_005690416	E2-E3	E4-E5
	*Ovis aries*	NM_001009327	E2-E3	E4-E5
**IL-12B**	*Bos taurus*	NM_174356	E3-E4	E4–E5
	*Capra hircus*	NM_001285700	E3-E4	E4–E5
	*Ovis aries*	NM_001009438	E3-E4	E4–E5
**INF-γ**	*Bos taurus*	NM_174086	E3	E3-E4
	*Capra hircus*	NM_001285682	E2-E3	E3
	*Ovis aries*	NM_001009803	E2-E3	E3-E4
**TNF-α**	*Bos taurus*	NM_173966	E1–E2	E3–E4
	*Capra hircus*	NM_001286442	E1–E2	E3–E4
	*Ovis aries*	NM_001024860	E1–E2	E3–E4
***GAPDH***	*Bos taurus*	NM_001034034	E2-3	E4-5
	*Capra hircus*	XM_005680968	E2-E3	E4-5
	*Ovis aries*	NM_001190390	E2-E3	E4-5
***H3F3A***	*Bos taurus*	NM_00101489	E2	E2-E3
	*Capra hircus*	XM_005690530	E1	E1-E2
	*Ovis aries*	XM_004013633	E2	E2-E3
***ACTB***	*Bos taurus*	NM_173979	E4-E5	E5-E6
	*Capra hircus*	XM_005694067	E3-E4	E4-E5
	*Ovis aries*	NM_001009784	E4-E5	E5-E6
***PPIA***	*Bos taurus*	NM_178320	E4	E4-E5
	*Capra hircus*	XM_005679322	E3	E3-E4
	*Ovis aries*	XM_004013990	-	-
***YWHAZ***	*Bos taurus*	NM_174814	E3-E4	E4-E5
	*Capra hircus*	XM_005689196	E3-E4	E4-E5
	*Ovis aries*	NM_001267887	E2-E3	E3-E4

Abbreviations. F: forward; R: reverse; bp: base pair; IL, interleukin; IL12B, interleukin p40; TNF-α tumor necrosis factor-alpha; IFN-γ interferon-gamma; ACTB, beta*-*actin; GAPDH, Glyceraldehyde 3-phosphate dehydrogenase; H3F3A, H3-histone family 3A; PPIA, peptidylprolyl isomerase A; YWHAZ, tyrosine 3-monooxygenase/tryptophan 5-monooxygenase activation protein, zeta polypeptide;

^a^: GenBank accession number.

**Table 2:** **Primer characteristics of cytokine and reference genes for bovine, caprine and ovine gene expression**

**Target gene**	**Primer sequence (5′-3′)**	**Optimal primer concentration (nM)**	**Amplicon size (bp)**
**IL-4**	F-CAGCATGGAGCTGCCT	300	177
	R-ACAGAACAGGTCTTGCTTGC	300	
**IL-10**	F-CTTTAAGGGTTACCTGGGTTGC	300	239
	R-CTCACTCATGGCTTTGTAGACAC	300	
**IL-12B**	F-CAGCAGAGGCTCCTCTGAC	600	237
	R-GTCTGGTTTGATGATGTCCCTG	600	
**INF-γ**	F-CAGAGCCAAATTGTCTCCTTC	300	167
	R-ATCCACCGGAATTTGAATCAG	300	
**TNF-α**	F-CCAGAGGGAAGAGCAGTCC	300	111
	R-GGCTACAACGTGGGCTACC	300	
***GAPDH***	F–ATCTCGCTCCTGGAAGATG	600	227
	R-TCGGAGTGAACGGATTCG	300	
***H3F3A***	F-GAGGTCTCTATACCATGGCTC	300	150
	R-GTACCAGGCCTGTAACGATG	300	
***ACTB***	F-TGGGCATGGAATCCTG	600	194
	R-GGCGCGATGATCTTGAT	600	
***PPIA***	F-TGACTTCACACGCCATAAT	300	180
	R-CTTGCCATCCAACCACTC	600	
***YWHAZ***	F-GAAAGGGATTGTGGACCAG	300	183
	R-GGCTTCATCAAATGCTGTCT	300	

Abbreviations. F: forward; R: reverse; bp: base pair; IL, interleukin; IL12B, interleukin p40; TNF-α tumor necrosis factor-alpha; IFN-γ interferon-gamma; ACTB, beta*-*actin; GAPDH, Glyceraldehyde 3-phosphate dehydrogenase; H3F3A, H3-histone family 3A; PPIA, peptidylprolyl isomerase A; YWHAZ, tyrosine 3-monooxygenase/tryptophan 5-monooxygenase activation protein, zeta polypeptide;

Amplicon sizes were determinate in *Bos taurus*. Amplicon sizes in *Capra hircus* and *Ovis aries* were similar.

### Checking specificity with conventional PCR assays

The specificity of each primer pair was checked in preliminary conventional PCR assays with bovine, ovine and caprine cDNA synthesized from Tempus™ total RNA. All PCR reactions were performed with SureStart Taq DNA Polymerase (Agilent Technologies, Inc. Santa Clara, USA) following the manufacturer’s instructions, with 10 ng of cDNA in 0.5 μM of each primer. PCR reactions were conducted on a SureCycler 8800 Thermal Cycler (Agilent Technologies, Santa Clara, USA). The PCR program consisted in an initial denaturation step of 10 min at 95°C, followed by 40 cycles of 30 sec at 95°C, 30 sec at 60°C, and 45 sec at 72°C, followed by a final elongation step of 10 min at 72°C. PCR products were analyzed on 2% agarose gel. Amplicon size was checked with agarose electrophoresis migration (amplicon sizes are listed in Table 2). Finally, PCR products were (i) purified with QIAquick PCR Purification Kit (Qiagen Ltd., Crawley, UK), according to the manufacturer’s instructions, (ii) quantified using a NanoDrop™ ND-1000 Spectrophotometer (Thermo Fisher Scientific, MA, USA), and (iii) sequenced (Beckman Coulter Genomics, Takeley, UK).

The absence of genomic DNA (gDNA) amplification was checked with bovine, ovine and caprine gDNA isolated from blood buffy coat, using the QIAamp DNA Mini Kit (Qiagen Ltd., Crawley, UK).

### Quantitative PCR assays

Quantitative PCR (QPCR) reactions were conducted on Mx3005P QPCR Systems™ (Agilent Technologies, Santa Clara, USA). Amplifications were performed with Brilliant SYBR® II QPCR Master kit (Agilent Technologies, Santa Clara, USA). Following the manufacturer’s instructions, reactions were carried out in a final volume of 25 μl with 1.5 μl of cDNA and 1 μl of each primer at the optimized concentration. Amplifications were performed as described above for conventional PCR assays. A dissociation step was included for all reactions to confirm single specific PCR product amplification and define the Tm of each amplicon. A negative control (no-template control) was included in each primer assay to check for the formation of primer-dimers.

### Linearity and efficiency of the qPCR reaction

QPCR reaction efficiency (E) was determined for each purified and quantified PCR product by performing a 10-fold serial dilution in eight points, in duplicate. Calibration curves were plotted; efficiencies and correlation coefficients were calculated by MxPro QPCR Software (Agilent Technologies, Santa Clara, USA).

### Absolute quantification and relative expression genes in Tempus™ and PBMC samples

The RT-qPCR method was checked by amplification, in duplicate, of cDNA synthesized from (i) Tempus™ total RNA from three different animals (absolute quantification), and (ii) PBMC (ConcanavalinA-stimulated and unstimulated) total RNA from three independent experiments (relative expression).

Absolute quantification results are expressed as the mean number of copies (nc) from six amplification values with standard deviation. Nc was calculated using the following equation: Nc=(Relative amount of target (in ng) × 10^−9^)/(DNA length(dp) x 650) × 6.022 × 10^23^. The relative amount of target (in ng) was calculated using the calibration curve and the following equation: Relative amount of target (in ng)=10 ((number of Cq-intercept)/slope).

Relative expression was calculated with efficiency correction using the relative expression software tool (REST) [30]. Multiple reference gene normalization was chosen.

Gene expression stability of the five candidate reference genes was determined in PBMC samples and calculated using the geNorm application in Microsoft Excel [23]. Stability values (*M*-value) and pairwise variations (*V*-score) were calculated and the optimal number of reference genes required for relative expression was determined. In addition, the best stable combination of reference genes was selected with NormFinder Excel Add-In [31]. The relative expression ratio (Er) of cytokine genes in ConcanavalinA-stimulated cells (n=3) compared to unstimulated cells (n=3) was calculated using the quantification of cytokine and reference gene as number of copies. Expression variation for each cytokine gene was provided by REST [30].

### Stability of the five candidate reference genes using geNorm and NormFinder analysis

The gene expression stability of the five candidate reference genes was determined in Tempus™ and PBMC samples and calculated with two different reference gene selection algorithms. The geNorm application in Microsoft Excel [23] and NormFinder Excell Add-In [31] calculate stability values (*M*-value in geNorm and ρ-value in NormFinder) and the reference gene with the lowest stability value is the most stable expression. NormFinder identify the best stable combination of reference genes.

As recommended by the authors of the geNorm method, an *M*-value <1.5 is the cut-off for suitability as reference genes for PBMC samples (heterogeneous samples) and *M*-value <0.5 for Tempus™ samples (homogeneous samples). In addition, using the geNorm application, we calculated the pairwise variation (*V*-score) to determine the optimal number of reference genes required using a *V*-value <0.25 as a cut-off for suitable reference genes in PBMC samples (heterogeneous samples) and *V*-value <0.15 in Tempus™ samples (homogeneous samples). All analyses were performed using the quantification of cytokine and reference genes as number of copies. Data were analyzed by NormFinder with groups identified (unstimulated and stimulated group).

### Reproducibility and sensitivity of the RT-qPCR method

Inter-assay variability (R) is the variation in amplification results between different runs for each sample. R was determined by amplification of cDNA synthesized from the Tempus™ total RNA, in duplicate, repeated in three independent runs. R is expressed as Cq and as the mean number of copies (nc) with standard deviation.

The limit of detection (LOD) was the highest to the lowest quantifiable number of copies performed after serial dilutions of the cDNA pool synthesized from the Tempus™ total RNA. LOD is expressed as nc.

## Results

### Primer design and control of primer specificity

Cytokine and reference gene primer sets were designed jointly for the three ruminant species, cattle, sheep and goats, according to the predefined parameters described in “Methods”. The characteristics of the primer sets are listed in Table 2. All primer sets allowed specific hybridization and amplification at 60°C. Products were checked by electrophoresis migration and led to a single specific amplicon of the expected size. Also, the absence of amplification with bovine genomic DNA was confirmed.

In addition, similarities between the sequenced PCR products and the corresponding target on the mRNA reference sequences (*Bos taurus, Capra hircus*, *Ovis aries*) from the NCBI GenBank database were checked using the standard nucleotide BLAST program [32]. To sum up, all qPCR amplifications led to a single specific peak and confirmed the amplification of a single specific product.

### Linearity and efficiency

The optimal concentration of each primer was determined as the lowest concentration of the reverse and forward PCR primers that resulted in the lowest Cq with no formation of primer-dimer. The absence of no-specific products and primer-dimers was confirmed. Concentrations of primers are listed in Table 2. Single-peak melting curves of the PCR products defined the melting temperatures of PCR products (see Table 3 and Table 4). No amplification was detected in no-RT controls and no-template controls.

**Table 3:** **Assay performances for five cytokine genes for bovine, caprine and ovine gene expression**

**Target gene**	**Species**	**Tm (°C)**	**Intercept**	**Slope**	**E (%)**	**Inter-assay variability**				**LOD nc**
						**Mean Cq**	**SD Cq**	**Mean nc**	**SD nc**	
**IL-4**	cattle	83.9	2.02	−3.372	102	28.5	0.2	25	3	9
	goat		1.37	−3.276	99	28.6	0.2	30	4	11
	sheep		1.39	−3.283	99	28.1	0.1	38	2	5
**IL-10**	cattle	86.6	0.27	−3.50	95	27.6	0.2	104	16	6
	goat		0.91	−3.33	97	28.9	0.2	30	4	7
	sheep		1.12	−3.34	101	29.2	0.2	17	2	5
**IL-12B**	cattle	86.5	0.8	−3.32	97	27.3	0.4	95	8	1
	goat		1.70	−3.28	95	35	0.6	<1	-	2
	sheep		1.59	−3.31	95	33	0.5	2	0.6	5
**INF-** **γ**	cattle	80.2	1.27	−3.449	95	27.8	0.0	79	2	5
	goat		1.63	−3.309	95	28.3	0.1	68	5	6
	sheep		1.02	−3.409	96	27.2	0.2	128	18	2
**TNF-** **α**	cattle	84.5	2.18	−3.417	91	25.0	0.3	837	192	2
	goat		0.38	−3.350	99	25.1	0.1	387	35	3
	sheep		0.04	−3.350	97	25.7	0.2	307	35	1

Abbreviations. E, reaction efficiency; Tm, melting temperature; SD: Standard deviation; LOD, limit of detection; nc: number of copies. Abbreviations for cytokine genes see Table 1.

Inter-assay variability (R) is the variation in amplification results between different runs for each sample. R was determined with amplification of cDNA synthesized from Tempus™ whole blood total RNA, in duplicate, repeated in three independent runs. R is expressed in Cq and mean number of copies (nc) with standard deviation (SD).

Limit of detection (LOD) is the lowest number of copies correctly amplified, quantified that led to single specific product amplification at the expected Tm after serial dilutions of cDNA pool synthesized from the Tempus^TM^ total RNA. Nc was calculated using the following equation: Nc=(Relative amount of target (in ng) × 10^−9^)/(DNA length (dp) x 650) × 6.022 × 10^23^. Relative amount of target (in ng) was calculated using the calibration curve and the following equation: Relative amount of target (in ng)=10 ((number of Cq-intercept)/slope).

**Table 4:** **Assay performances for reference genes for bovine, caprine and ovine gene expression**

**Target gene**	**Species**	**Tm (°C)**	**Intercept**	**Slope**	**E (%)**	**Inter-assay variability**				**LOD nc**
						**Mean Cq**	**SD Cq**	**Mean nc**	**SD nc**	
***GAPDH***	cattle	84.5	2.02	−3.372	98	18.3	0.2	6.2 10^4^	0.7 10^4^	1
	goat		1.37	−3.276	102	19.6	0.2	1.1 10^4^	0.2 10^4^	1
	sheep		1.39	−3.283	102	24.9	0.1	285	12	2
***H3F3A***	cattle	85	0.27	−3.50	93	16.8	0.1	11.4 10^4^	0.5 10^4^	20
	goat		0.91	−3.33	100	16.4	0.1	14.2 10^4^	0.7 10^4^	11
	sheep		1.12	−3.34	99	16.5	0.1	15.4 10^4^	1.1 10^4^	14
***ACTB***	cattle	85.7	0.8	−3.32	100	15.4	0.4	19.4 10^4^	5.1 10^4^	8
	goat		1.70	−3.28	102	15.7	0.2	25.6 10^4^	3.8 10^4^	1
	sheep		1.59	−3.31	101	15.6	0.3	27.8 10^4^	0.5 10^4^	6
***PPIA***	cattle	82.3	1.27	−3.449	95	17.9	0.1	7.7 10^4^	0.5 10^4^	6
	goat		1.63	−3.309	100	17.5	0.1	8.3 10^4^	0.6 10^4^	3
	sheep		1.02	−3.409	96	17.4	0.1	8.2 10^4^	0.5 10^4^	8
***YWHAZ***	cattle	81.3	2.18	−3.417	96	18.7	0.1	7.2 10^4^	0.7 10^4^	9
	goat		0.38	−3.350	99	23.8	0.1	506	33	4
	sheep		0.04	−3.350	99	23.5	0.1	506	24	3

Abbreviations and notes, see Table 3.

All calibration curves produced a linear standard curve and efficiency (E) ranged from 91% (for bovine TNF-α) to 102% (bovine IL-4, ovine and caprine GAPDH, caprine ACTB). All correlation coefficients were higher than 0.998 and confirmed the reaction linearity of all primer sets. PCR efficiency, the slopes and intercepts of calibration curves are listed in Table 3 (cytokine genes) and Table 4 (reference genes). Representative dissociation and calibration curves for the three species are presented in Additional file 1 (cytokine genes) and in Additional file 2 (reference genes).

### Reproducibility and sensitivity

To evaluate the reproducibility of our qPCR assays, we performed amplification of Tempus™ samples (whole blood samples). All genes were correctly amplified whatever the species and led to single specific product amplification at the expected Tm. Inter-assay variability was calculated using the standard deviation of the six amplification values (Cq and copy values). Variability of cytokine gene amplifications ranged between 0.0 Cq (bovine INF-γ, corresponding to 2 copies) and 0.4 Cq (bovine IL-12B, corresponding to 8 copies). Variability of reference gene amplifications ranged between 0.1 Cq (caprine YWHAZ, corresponding to 33 copies) and 0.4 Cq (bovine ACTB, corresponding to 5.1 10^4^ copies). Inter-assay variability is listed in Table 3 (cytokine genes) and Table 4 (reference genes).

Finally, we evaluated the lowest number of correctly amplified and quantified copies that led to single specific product amplification at the expected Tm (LOD) with our assay. In bovine samples, the lowest quantifiable number of copies was one copy both cytokine genes (IL-12B) and reference genes (GAPDH). In caprine samples, the lowest quantifiable number of copies was two copies for cytokine genes (IL-12B) and one copy for reference genes (GAPDH and ACTB). In ovine samples, the lowest quantifiable number of copies was one copy for cytokine genes (TNF-α) and two copies for reference genes (GAPDH). LOD are listed in Table 3 (cytokine genes) and Table 4 (reference genes).

### Validation of the RT-qPCR method: absolute quantification of cytokine and reference gene expression

Absolute quantification was checked by amplification, in duplicate, of cDNA synthesized from Tempus™ total RNA (whole blood samples) from three different animals of each species. All genes were correctly amplified whatever the species, and led to a single specific product amplification at the expected Tm. The mean number of copies and Cq with SD from the six amplification values are listed in Table 5.

**Table 5:** **Absolute quantification of cytokine and reference gene expression in whole blood samples**

**Target gene**	**Species**	**Cq values**		**Copie number values**	
		**Mean Cq**	**SD Cq**	**Mean nc**	**SD nc**
**IL-4**	cattle	28.7	0.3	24	5
	goat	28.5	0.2	31	3
	sheep	28.1	0.5	39	12
**IL-10**	cattle	27.2	0.6	156	54
	goat	28.2	0.5	48	17
	sheep	28.5	0.1	28	1
**IL-12B**	cattle	27.9	0.6	62	21
	goat	34.9	1.2	2	0.4
	sheep	33.3	0.5	2	0.7
**INF-**γ	cattle	27.4	0.4	106	28
	goat	27.7	0.3	103	21
	sheep	26.3	0.3	241	52
**TNF-**α	cattle	24.7	0.4	965	199
	goat	24.0	0.1	978	34
	sheep	24.4	0.1	728	34
***GAPDH***	cattle	18.0	0.3	7.6 10^4^	1.2 10^4^
	goat	19.1	0.1	1.6 10^4^	0.1 10^4^
	sheep	25.0	0.2	264	38
***H3F3A***	cattle	16.7	0.1	12.5 10^4^	0.7 10^4^
	goat	16.3	0.2	14.9 10^4^	2.3 10^4^
	sheep	16.2	0.1	19.4 10^4^	1.6 10^4^
***ACTB***	cattle	15.1	0.3	24.5 10^4^	4.3 10^4^
	goat	15.4	0.1	32.9 10^4^	2.8 10^4^
	sheep	15.5	0.2	30.2 10^4^	4.6 10^4^
***PPIA***	cattle	18.4	0.2	58.0 10^4^	0.6 10^4^
	goat	17.7	0.2	75.6 10^4^	0.8 10^4^
	sheep	17.7	0.1	65.8 10^4^	0.4 10^4^
***YWHAZ***	cattle	18.8	0.5	70.9 10^4^	1.9 10^4^
	goat	23.5	0.3	647	120
	sheep	23.6	0.2	482	81

Abbreviations for cytokine and reference genes, see Table 1.

Absolute quantification was checked by amplification of cDNA synthesized from Tempus^TM^ total RNA from three different animals of each species. Absolute quantification used the standard curve method and is expressed as the number of copies (nc) and Cq with standard deviation (SD) from six amplification values.

In bovine samples, the number of cytokine gene copies detected was between 24 +/–5 copies (IL-4, corresponding to 28.7 +/–0.3 Cq) and 965 +/–199 copies (TNF-α, corresponding to 24.7 +/–0.3 Cq). In ovine and caprine samples, the number of cytokine gene copies detected was similar: between 2 +/–0.7 copies (ovine IL-12B, corresponding 33.3 +/–0.5 Cq) and 798 +/–34 copies (caprine TNF-α, corresponding to 24.0 +/–0.1 Cq).

GAPDH was the most weakly expressed reference gene with 264 +/–38 copies (ovine GAPDH, corresponding to 25.0 +/–0.2 Cq) and PPIA was the most strongly expressed reference gene with 75.6 10^4^ +/–0.8 10^4^ copies (caprine PPIA, corresponding to 17.7 +/–0.2 Cq). H3F3A and ACTB were expressed almost identically in the three species with, for example, respectively 12.5 10^4^ +/–0.7 10^4^ copies in bovine samples (corresponding to 16.7 +/–0.1 Cq) and 30.2 10^4^ +/–4.6 10^4^ copies in ovine samples (corresponding to 15.5 +/–0.2 Cq). Surprisingly, the level of GAPDH and YWHAZ was differently expressed in the three species. GAPDH was weakly expressed in ovine samples with 264 +/–38 copies (corresponding to 25.0 +/–0.2 Cq) but strongly expressed in bovine and caprine samples with 7.6 10^4^ +/–1.2 10^4^ copies (corresponding to 18.0 +/–0.3 Cq). YWHAZ was weakly expressed in ovine and caprine samples with 482 +/–81 copies (ovine, corresponding to 23.6 +/–0.2 Cq) but strongly expressed in bovine samples with 70.9 10^4^ +/–1.9 10^4^ copies (corresponding to 18.8 +/–0.5 Cq). These differences in expression gene levels are consistent with the levels observed in unstimulated PBMC assays (see Additional file 3).

### Validation of the RT-qPCR method: relative expression of cytokine genes in stimulated PBMC samples

**Figure 1:**
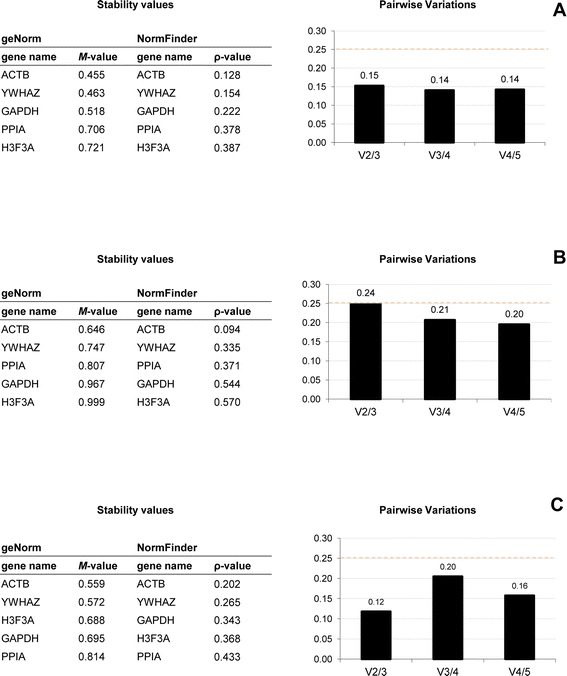
**Stability of five candidate reference genes in PBMC samples using geNorm and NormFinder analysis.** Abbreviations. PBMC, peripheral blood mononuclear cells. Abbreviations for reference genes, see Table 1. PBMCs were cultured with medium (unstimulated condition) or stimulated with 5 μg/ml of Concanavalin A (stimulated condition) for 36 h. Stability of the five reference genes were calculated in bovine **(A)**, caprine **(B)** and ovine **(C)** PBMC samples using geNorm application and NormFinder software. Stability values were calculated as *M*-value in geNorm and ρ-value in NormFinder. The reference genes were presented in ranked list of the most stable gene to the least stable gene. The pairwise variation (*V*-score) was performed with geNorm application to determine the optimal number of required reference genes for relative expression in bovine **(A)**, caprine **(B)** and ovine **(C)** PBMC samples.

The relative expression of ConcanavalinA-stimulated PBMC samples compared with unstimulated PBMC samples in three independent experiments for each species was calculated using the expression level of cytokine and reference genes. The stability results are presented in Figure 1. Quantification of cytokine and reference genes in unstimulated and ConcanavalinA-stimulated PBMC samples are presented in Additional file 3.

First, the optimal number of reference genes required for relative expression was determined using the geNorm application. Based on the cut-off pairwise variation of 0.25 (*V*-score), in our study, the use of two reference genes was sufficient for accurate normalization whatever the species, with a *V*-score of between 0.12 (pairwise variation V2/3 in ovine PBMC samples) and 0.24 (pairwise variation V2/3 in caprine PBMC samples). Second, the best stable combination of two reference genes was identified by NormFinder analysis: PPIA/H3F3A genes for bovine samples (with a stability value of 0.024), GAPDH/H3F3A genes for caprine samples (with a stability value of 0.072) and PPIA/H3F3A genes for ovine samples (with a stability value of 0.064). Each combination of reference genes was used to calculate the relative expression of cytokine genes.

**Figure 2:**
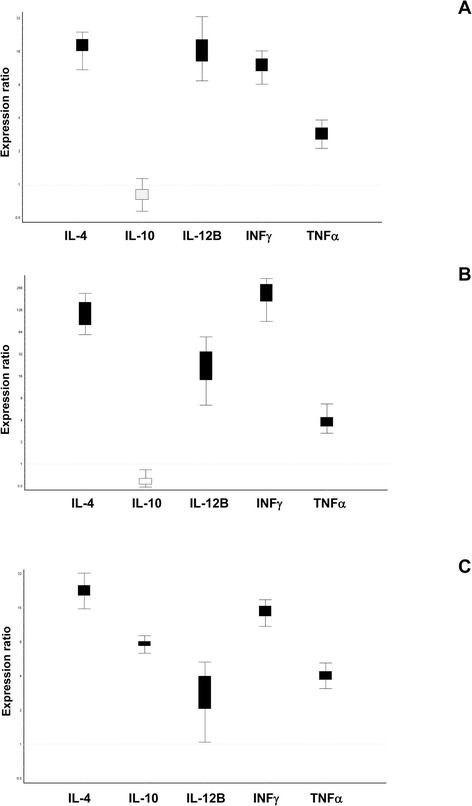
**Cytokine relative expression in bovine, caprine and ovine stimulated PBMC samples.** Abbreviations. PBMC, peripheral blood mononuclear cells. Abbreviations for reference genes, see Table 1. PBMCs were cultured with medium (unstimulated condition) or stimulated with 5 μg/ml of Concanavalin A (stimulated condition) for 36 h. The relative expression ratio (Er) of cytokine genes in Concanavalin A-stimulated cells compared to unstimulated cells was calculated (amplification of three independent experiments in duplicate) in bovine **(A)**, caprine **(B)** and ovine **(C)** samples using the relative expression software tool (REST) with the most stable combination of two reference genes identified with NormFinder software. REST Software uses randomization technique and error bars represents distribution of permutated expression data. Black boxes represent up-regulated cytokine expression and white boxes down-regulated cytokine expression.

Finally, a qPCR assay enabled the relative expression of cytokine to be determined in all samples, whatever the species, even in cases of strong up-regulation of cytokine mRNA expression (i.e. caprine INF-γ) or in cases of decreased expression of cytokine mRNA (i.e. bovine IL-10). Cytokine expression results are presented in the whisker-box plots provided by REST [30] (see Figure 2).

### Stability of the five reference genes and comparison between geNorm and NormFinder analyses and samples

**Figure 3:**
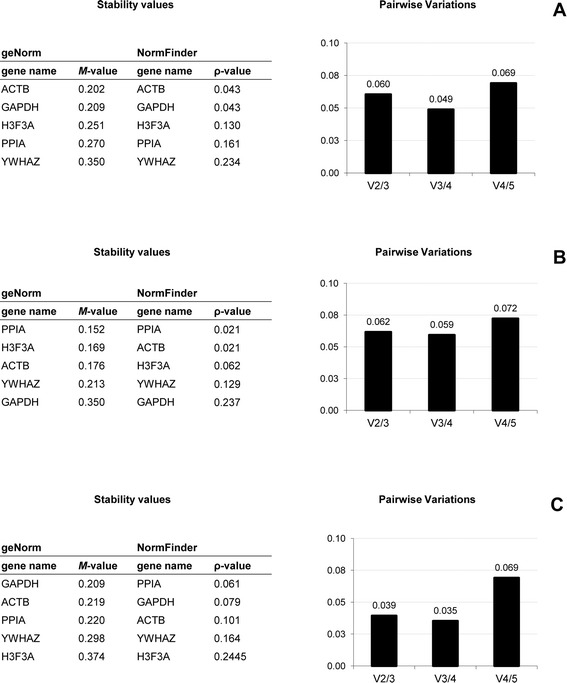
**Stability of five candidate reference genes in whole blood samples using geNorm and NormFinder analysis.** Abbreviations for reference genes, see Table 1. Whole blood (or Tempus™) samples were performed from three different animals of each species. Stability of the five reference genes were calculated in bovine **(A)**, caprine **(B)** and ovine **(C)** whole blood samples using geNorm application and NormFinder software. Stability values were calculated as *M*-value in geNorm and ρ-value in NormFinder. The reference genes were presented in ranked list of the most stable gene to the least stable gene. The pairwise variation (*V*-score) was performed with geNorm application to determine the optimal number of required reference genes for relative expression in bovine **(A)**, caprine **(B)** and ovine **(C)** whole blood samples.

The expression of the five selected reference genes (GAPDH, ACTB, YWHAZ, PPIA and H3F3A) was determined in all samples, in both PBMC and Tempus™ (whole blood) samples, using two statistical approaches, geNorm application [23] and NormFinder software [31]. Figure 1 shows the ranked lists of genes and pairwise variations for the PBMC samples and Figure 3, for the whole blood samples.

In the PBMC samples, *M*-values were lower than 1.5 and between 0.455 (bovine ACTB) and 0.999 (ovine H3F3A), and *V*-values were lower than 0.25 and between 0.12 (caprine V2/3) and 0.24 (ovine V2/3). In whole blood samples, *M*-values were lower than 0.5 and between 0.152 (caprine PPIA) and 0.374 (ovine H3F3A), and *V*-values were lower than 0.15 and between 0.039 (caprine V2/3) and 0.062 (ovine V2/3).

In bovine samples, the most stable reference gene in both whole blood and PBMC samples was ACTB, whatever the analysis. Also, the ranked list of reference genes, provided by both software, was the same for PBMC and for whole blood samples.

In ovine samples, stability results were very similar. For PBMC samples, the ranked lists of reference genes, produced by geNorm and NormFinder were the same and the most stable reference gene was ACTB. In whole blood samples, the three most stable genes were GAPDH, ACTB and PPIA, with very similar stability values (0.209 < *M*-value < 0.220).

In caprine samples, the ranked lists of reference genes for samples and analyses differed. In PBMC samples, ACTB and YWHAZ were the two most stable reference genes according to both geNorm and NormFinder analyses. In the whole blood samples, PPIA was the most stable reference gene and the three most stable genes were the same, with very close stability values (0.152 < *M*-value < 0.176).

## Discussion

We developed a novel SYBR Green RT-qPCR assay for the simultaneous relative quantification of five major cytokines (IL-4, IL-10, IL-12B, IFN-γ and TNF-α) in cattle, sheep and goats, and accurate normalization with five candidate reference genes (GAPDH, ACTB, H3F3A, PPIA and YWHAZ). The main advantage of this original work is the design of an optimal primer set for three ruminant species with the same hybridization temperature. To our knowledge, this is the first SYBR Green assay that enables quantification of bovine, ovine and caprine cytokines with a single set of reagents using the same protocol. Due to the lack of tools to investigate host immunity by quantification of secreted cytokines, analysis of cytokine gene expression has become a widely used method to establish a cytokine profile in ruminants. Many infectious diseases (for example Johne’s disease) affect many animal species and threaten livestock especially in the case of multi-species grazing. Our assay could thus greatly improve research by scientists involved in ruminant diseases, particularly to characterize the immune response to pathogens affecting cattle, sheep, and goats.

RT-qPCR is one of the most widely used methods to investigate cytokine gene expression in ruminants thanks to its high specificity, sensitivity and accuracy. It is also the most suitable for expression analysis of miscellaneous cytokines from a single sample and a small quantity of template material. However, to ensure accurate reproducible quantitative data, strict standard operating procedures have to be followed and many reviews have demonstrated the importance of the RT-qPCR workflow [21,33,34].

For that reason, we developed the novel SYBR Green RT-qPCR assay presented here taking particular care to maximize the assay, detailing each step of the experimental protocol, from primer design, sample preparation or reverse transcription to the choice and normalization by reference genes, and reporting on the performance of the assays.

We first focused our attention on the criteria needed for primer design, particularly primer location straddling exons, and the choice of a single hybridization temperature. S. Taylor [33] underlines the importance of primer design and the choice of target sequence to ensure specific efficient amplification of the products. Indeed, a design that spans exon*-*exon boundaries of at least one of the two primers, in addition to the DNAse treatment, prevents amplification of the target from contaminating gDNA. In our study, all primers were carried out fulfilling our criteria-with the exception of five primers that could not be designed to span exon*-*exon boundaries-to achieve a GC% of between 40% and 70% and a melting temperature of between 62°C and 65°C. This was the case for forward primers of H3F3A, PPIA, IL-4, and INF-γ genes, and for the reverse primer of the IL-10 gene. In these cases, the second primer was then designed to span exon*-*exon boundaries. Thus, absence of amplification in no-RT controls and with genomic DNA, reaction linearity higher than 0.998 and high PCR efficiencies of all primer sets helped design sound primers and ensured that contaminating gDNA amplification did not occur.

Using the same hybridization temperature made it possible to analyze many samples in the same run, to compare the level of expression of a particular gene between different samples and to perform simultaneous normalization. With the common primer design, the same hybridization temperature had the additional advantage of enabling different samples of three ruminant species to be analyzed in the same run. This reduced the time and cost of the analysis, and also improved reproducibility, as demonstrated by our low run-to-run variation.

We then carefully selected five reference genes, the most widely used (GAPDH and ACTB) and/or those reported to be the most stable, for accurate normalization in both different types of samples and in the three target species. Accurate normalization is essential to eliminate sample-to-sample variations and to obtain reliable results, particularly in relative gene expression studies [21] for which multiple normalization genes with three to five reference genes could be required [23].

Before selecting suitable reference genes for normalization, the stability of the candidate reference genes (GAPDH, ACTB, H3F3A, PPIA and YWHAZ) has to be confirmed and the optimal number of reference genes required for an accurate relative expression has to be determined. In fact, there is no consensus on which method should be used to examine reference gene expression stability. We chose to evaluate the gene expression stability of the five reference genes using geNorm application [23] and NormFinder software [31] as two complementary statistical approaches. We showed that the most stable genes were similar or very close with the geNorm application and NormFinder software. In whole blood and PBMC assays, stability analyses allowed confirmation of the stability of the five candidate reference genes, which two were sufficient for an accurate normalization of our assays. While many studies recommend multiple normalization genes with three to five reference genes [33], we performed new REST analyses and compared the results of cytokine expression using the best combination genes selected by NormFinder software and the two or three most stable genes selected by geNorm application. Our results showed that gene expression was similar whatever the combination of genes selected (see results in Additional file 4), suggesting that a panel of five reference genes could be sufficient and underlining the importance of selecting adequate reference genes and checking their stability. In addition, differences and similarities in the expression stability of the reference genes were compiled. In our ovine PBMC assay, ACTB, YWHAZ and PPIA were the three most stable genes. These results were confirmed in our whole blood assay, where ACTB and PPIA were two of the three most stable reference genes. Several studies reported YWHAZ to be the most stable reference gene in ovine samples [27,35-37]. In addition to the above mentioned results, in our study, ACTB was also the most stable reference genes in bovine PBMC and whole blood samples, followed by YWHAZ and GAPDH. These results are partially in agree with Sheridan’s results using whole blood samples and with Spalenza and Robinson’s results using PBMC samples [24-26]. Our results emphasize the importance of following strict standard operating procedures and maximizing the assay to prevent technical variations. Finally, our results demonstrate the advantages of our RT-qPCR workflow, especially in the case of limited sample availability and funds.

In addition, we revealed an original difference in the expression level of GAPDH and YWHAZ genes among the three species, in both unstimulated PBMC and in whole blood samples. GAPDH and YWHAZ are involved in two different biological processes and have sophisticated functions in organisms in which their expression profile might fluctuate. GAPDH is an important enzyme for energy metabolism while YWHAZ plays a role in protein folding. The absence of any correlation between them in the variations we observed could exclude a GAPDH/YWHAZ co-regulation.

Our results emphasize the need to select different reference genes for accurate normalization, i.e. the most stable gene, or better yet, the most stable gene combination.

We demonstrated the sensitivity of the method and the correct cytokine gene expression quantification. In the case of low level mRNA, up-regulation, or a decrease in expression genes, an efficient RT-qPCR must be able to assess the cytokine profile regardless of the status of the animal or of the experimental conditions. Our assay ensured the detection of low levels of cytokine mRNA up to one or two copies. This was the case, for example, for IL-12B expression. We observed a low level of IL-12B expression in bovine and ovine unstimulated PBMC samples, as previously reported by Weiss in bovine macrophages and by Budhia in ovine PBMC [38]. Also, despite the low levels of IL-12B expression, 3 +/–1 copies and 8 +/−2 copies were obtained in bovine and ovine unstimulated PBMC samples respectively, up-regulation was determined relative to bovine and ovine stimulated PBMC samples by REST.

Relative expression results showed that IL-10 is up-regulated in ovine stimulated PBMC and not or only weakly down-regulated in bovine and caprine stimulated PBMC. We are unable to explain this difference in expression genes between species but we can compare with IL-12 expression, which was more strongly up-regulated in bovine and caprine stimulated PBMC.

Our SYBR Green RT-qPCR assay and the method we used to develop it, from primer design, through sample preparation and including validation, could be used to develop new cytokine primer sets and to increase the panel of cytokines and reference genes. These reference genes will also be useful for any gene expression studies whatever the species of ruminant.

## Conclusions

We developed a novel SYBR Green RT-qPCR assay for the simultaneous relative quantification of five major cytokines (IL-4, IL-10, IL-12B, IFN-γ and TNF-α) in cattle, sheep and goats, and their accurate normalization with five candidate reference genes. This novel SYBR Green RT-qPCR assay, which we validated in whole blood and PBMC samples, carries out accurate and reproducible quantitative data and ensures the detection of low levels of cytokine mRNA. This original assay is a robust, efficient, cost-effective, widely accessible tool to identify immunological markers for infections in three species of ruminants, cattle, sheep and goats.

## Additional files

Additional file 1:
**Representative dissociation and calibration curves of qPCR for reference genes.** Dissociation curves were plotted by MxPro QPCR Software. Single-peak melting curves defined the melting temperatures of PCR products and confirmed the amplification of a single specific product. QPCR reaction efficiency was determined for each purified and quantified PCR product by performing a 10-fold serial dilution in eight points, in duplicate. Calibration curves were plotted by MxPro QPCR Software. Additional file 2:
**Representative dissociation and calibration curves of qPCR for cytokine genes.** Dissociation curves were plotted by MxPro QPCR Software. Single-peak melting curves defined the melting temperatures of PCR products and confirmed the amplification of a single specific product. QPCR reaction efficiency was determined for each purified and quantified PCR product by performing a 10-fold serial dilution in eight points, in duplicate. Calibration curves were plotted by MxPro QPCR Software. Additional file 3:
**Quantification of cytokine and reference gene expression in unstimulated/stimulated PBMC assays.** Abbreviations for cytokine and reference genes, see Table 1. Quantification of cytokine and reference gene expression was checked by amplification of cDNA synthesized from ConcanavalinA-stimulated and unstimulated PBMC total RNA from three independent experiments for each species. Quantification of gene expression used the standard curve method and is expressed as the number of copies (nc) and Cq with standard deviation (SD) from three amplification values. Additional file 4:
**Cytokine gene expression using three combinations of reference genes selected by NormFinder and geNorm analyses.** Abbreviations for cytokine and reference genes, see Table 1. The relative expression ratio of cytokine genes in Concanavalin A-stimulated cells compared to unstimulated cells was calculated (amplification of three independent experiments in duplicate) in bovine (A), caprine (B) and ovine (C) samples using the relative expression software tool (REST) using three different combinations of reference genes selected by geNorm application an NormFinder software.

## Abbreviations

RT-qPCR: Reverse transcriptase quantitative polymerase chain reaction
IL: Interleukin
IL-12B: Interleukin p40
TNF-α: Tumor necrosis factor-alpha
IFN-γ: Interferon-gamma
ACTB: Beta*-*actin
GAPDH: Glyceraldehyde 3-phosphate dehydrogenase
H3F3A: H3-histone family 3A
PPIA: Peptidylprolyl Isomerase A
YWHAZ: Tyrosine 3-monooxygenase/tryptophan 5-monooxygenase activation protein, zeta polypeptide
MIQE: Minimum Information for publication of Quantitative Real-time PCR Experiments
E: Reaction efficiency
PBMC: Peripheral blood mononuclear cells
REST: Relative expression software tool
Er: Relative expression ratio
