# Supplementary figures and images for: Design and evaluation of a unique SYBR Green real-time RT-PCR assay for quantification of five major cytokines in cattle, sheep and goats
Source: BMC Vet Res. 2015 Mar 17;11:65. doi: 10.1186/s12917-015-0382-0 (PMC4369058; doi:10.1186/s12917-015-0382-0)

GAPDH

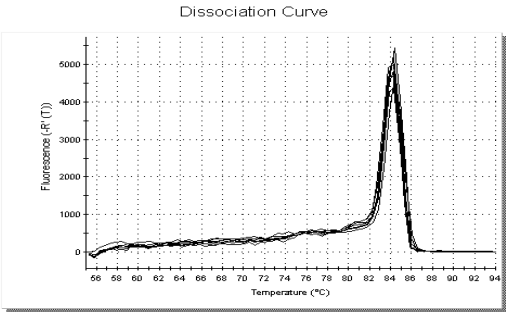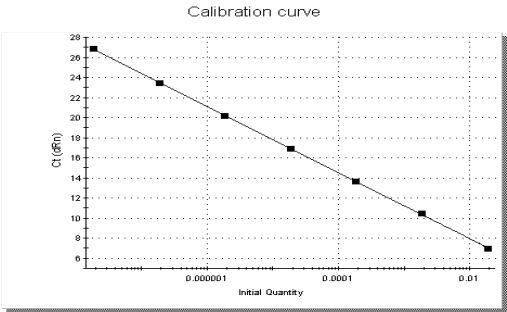

H3F3A

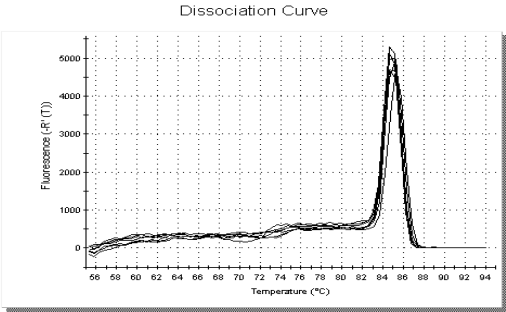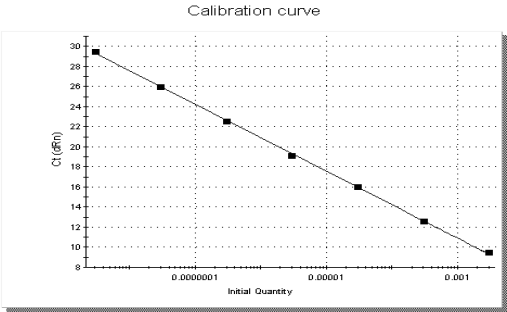

ACTB

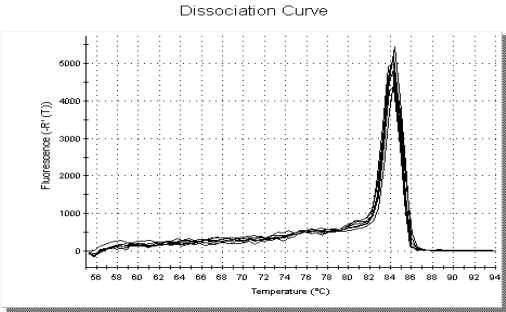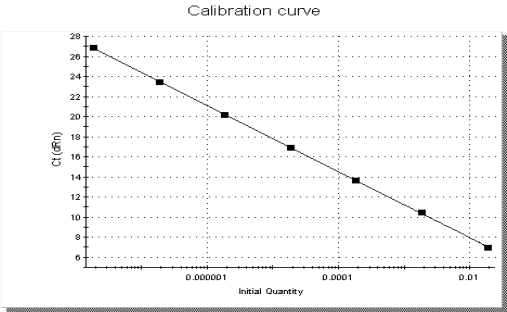

PPIA

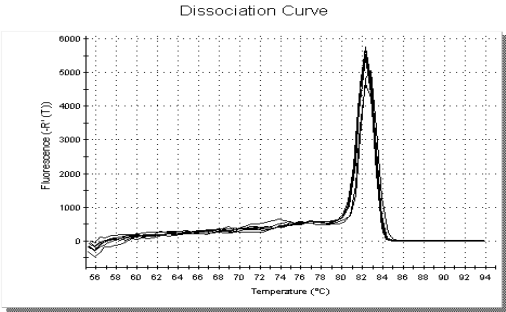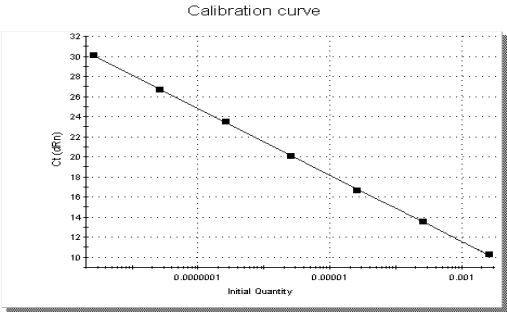

YWHAZ

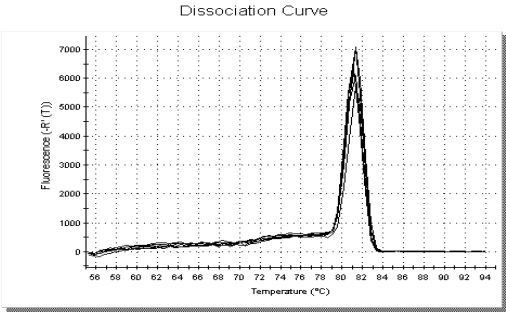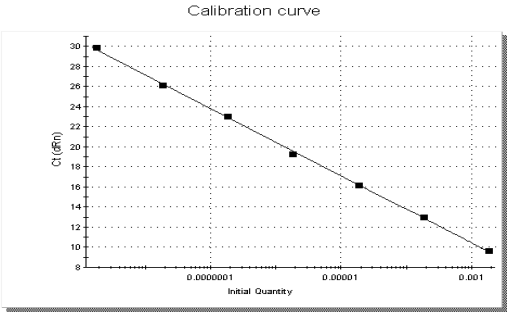

Supplement: Additional file 1: — Representative dissociation and calibration curves of qPCR for reference genes. Dissociation curves were plotted by MxPro QPCR Software. Single-peak melting curves defined the melting temperatures of PCR products and confirmed the amplification of a single specific product. QPCR reaction efficiency was determined for each purified and quantified PCR product by performing a 10-fold serial dilution in eight points, in duplicate. Calibration curves were plotted by MxPro QPCR Software. [file 12917_2015_382_MOESM1_ESM.pdf]

**IL-4**

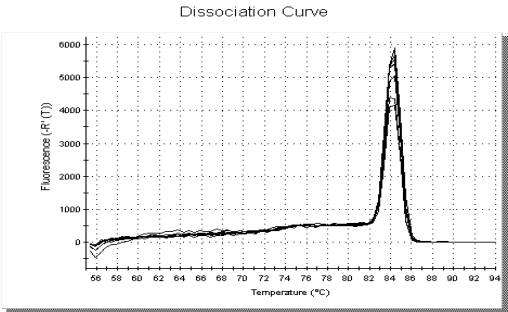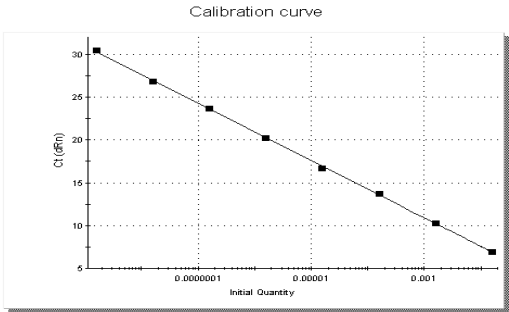

**IL-10**

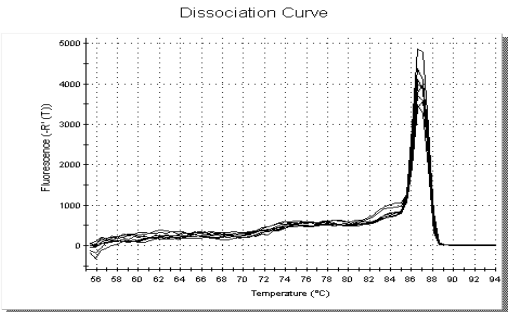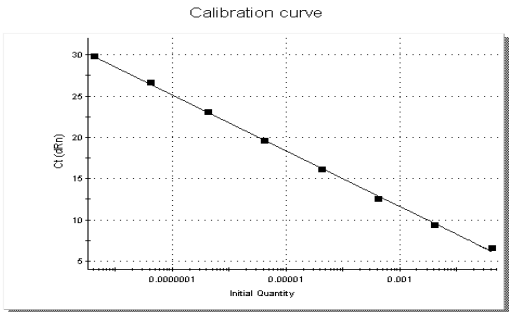

**IL-12B**

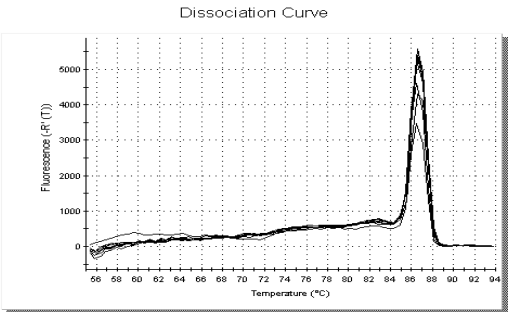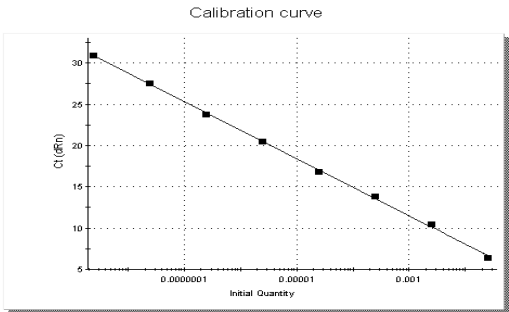

**INF<sub>γ</sub>**

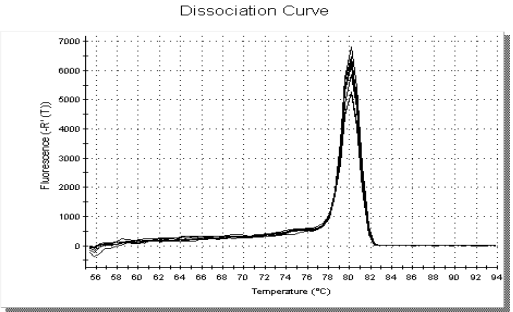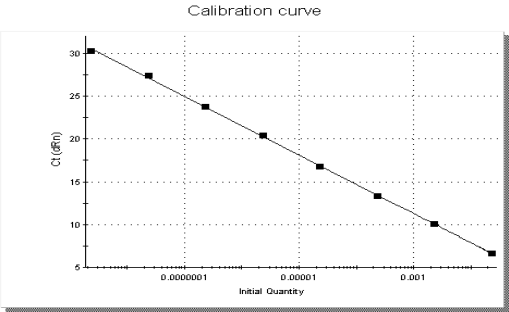

**TNF<sub>α</sub>**

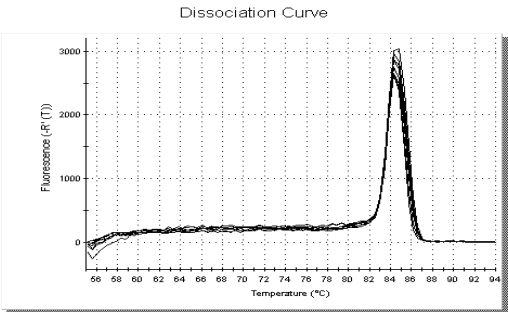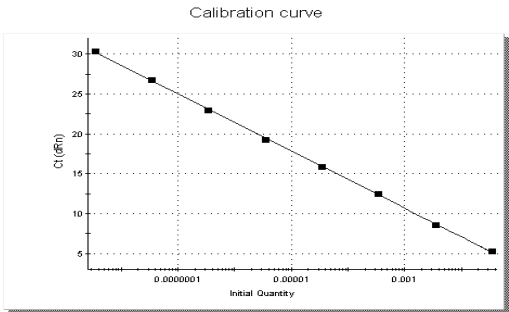

Supplement: Additional file 2: — Representative dissociation and calibration curves of qPCR for cytokine genes. Dissociation curves were plotted by MxPro QPCR Software. Single-peak melting curves defined the melting temperatures of PCR products and confirmed the amplification of a single specific product. QPCR reaction efficiency was determined for each purified and quantified PCR product by performing a 10-fold serial dilution in eight points, in duplicate. Calibration curves were plotted by MxPro QPCR Software. [file 12917_2015_382_MOESM2_ESM.pdf]
