# Supplementary material for: Design and evaluation of a unique SYBR Green real-time RT-PCR assay for quantification of five major cytokines in cattle, sheep and goats
Source: BMC Vet Res. 2015 Mar 17;11:65. doi: 10.1186/s12917-015-0382-0 (PMC4369058; doi:10.1186/s12917-015-0382-0)
Supplement: Additional file 3: — Quantification of cytokine and reference gene expression in unstimulated/stimulated PBMC assays. Abbreviations for cytokine and reference genes, see Table 1. Quantification of cytokine and reference gene expression was checked by amplification of cDNA synthesized from ConcanavalinA-stimulated and unstimulated PBMC total RNA from three independent experiments for each species. Quantification of gene expression used the standard curve method and is expressed as the number of copies (nc) and Cq with standard deviation (SD) from three amplification values. [file 12917_2015_382_MOESM3_ESM.pdf]

Additional files 3 – Quantification of cytokine and reference gene expression in unstimulated / stimulated PBMC assays

Target gene	species	unstimulated PBMC				stimulated PBMC			
		Cq values		Copie number values		Cq values		Copie number values	
		Mean Cq	SD Cq	Mean nc	SD nc	Mean Cq	SD Cq	Mean nc	SD nc
**IL-4**	cattle	28.9	0.2	20	3	23.4	0.2	963	152
	goat	28.9	0.3	24	5	20.4	0.3	8515	1828
	sheep	27.9	0.2	43	8	22.1	0.3	2357	535
**IL-10**	cattle	23.4	0.2	1788	190	22.3	0.3	3820	682
	goat	26.2	0.2	181	26	25.3	0.4	337	91
	sheep	25.7	0.1	199	20	21.5	0.2	3745	463
**IL-12B**	cattle	32.5	0.4	3	0	27.0	0.2	103	15
	goat	32.4	0.4	3.5	1	26.1	0.3	234	42
	sheep	31.3	0.5	8	2	28.6	0.6	52	19
**INFγ**	cattle	27.4	0.2	106	13	22.2	0.3	3334	643
	goat	24.7	0.1	730	55	15	0.1	47,6 10^{4}	2.8 10^{4}
	sheep	25.6	0.2	377	40	20.2	0.3	14 10^{3}	2.4 10^{3}
**TNFα**	cattle	25.3	0.2	642	85	22.2	0.1	4833	398
	goat	25.2	0.2	364	46	21.6	0.4	4434	1053
	sheep	25.3	0.0	410	12	21.9	0.1	3939	357
GAPDH	cattle	18.9	0.1	41.6 10^{3}	1.6 10^{3}	16.9	0.2	16.0 10^{4}	2.0 10^{4}
	goat	20.5	0.4	6260	1537	17.5	0.5	5.2 10^{4}	1.8 10^{4}
	sheep	25.8	0.3	149	25	23.9	0.2	590	105
H3F3A	cattle	17.0	0.1	10.3 10^{4}	0.4 10^{4}	16.6	0.1	13.4 10^{4}	0.6 10^{4}
	goat	16.6	0.2	11.8 10^{4}	1.9 10^{4}	16.3	0.2	15 10^{4}	2.1 10^{4}
	sheep	16.4	0.1	16.1 10^{4}	1.4 10^{4}	16.2	0.3	18.9 10^{4}	3.3 10^{4}
ACTB	cattle	15.8	0.1	14.7 10^{4}	1.1 10^{4}	14.6	0.2	35.0 10^{4}	6.3 10^{4}
	goat	16.7	0.3	12.8 10^{4}	2.8 10^{4}	15.3	0.4	35.6 10^{4}	10.3 10^{4}
	sheep	15.2	0.1	37 10^{4}	2.8 10^{4}	14.4	0.3	6.6 10^{5}	1.2 10^{5}
PPIA	cattle	18.3	0.1	60 10^{3}	2.3 10^{3}	15.8	0.1	32.1 10^{4}	2.4 10^{4}
	goat	18.5	0.3	40.6 10^{3}	7.5 10^{3}	15.9	0.4	26.0 10^{4}	7.8 10^{4}
	sheep	18.3	0.1	44 10^{3}	2.8 10^{3}	16.0	0.2	21 10^{4}	3.5 10^{4}
YWHAZ	cattle	19.2	0.0	54 10^{3}	1.5 10^{3}	18.1	0.2	11.0 10^{4}	1.3 10^{4}
	goat	24.6	0.3	3.5	68	23.7	0.3	575	106
	sheep	24.4	0.2	265	31	23.9	0.2	384	67
