# Supplementary material for: Design and evaluation of a unique SYBR Green real-time RT-PCR assay for quantification of five major cytokines in cattle, sheep and goats
Source: BMC Vet Res. 2015 Mar 17;11:65. doi: 10.1186/s12917-015-0382-0 (PMC4369058; doi:10.1186/s12917-015-0382-0)
Supplement: Additional file 4: — Cytokine gene expression using three combinations of reference genes selected by NormFinder and geNorm analyses. Abbreviations for cytokine and reference genes, see Table 1. The relative expression ratio of cytokine genes in Concanavalin A-stimulated cells compared to unstimulated cells was calculated (amplification of three independent experiments in duplicate) in bovine (A), caprine (B) and ovine (C) samples using the relative expression software tool (REST) using three different combinations of reference genes selected by geNorm application an NormFinder software. [file 12917_2015_382_MOESM4_ESM.pdf]

A

Expression ratios

**gene name**	**geNorm**		**NormFinder**
	**ACTB/YWHAZ**	**ACTB/YWHAZ/ GAPDH**	**PPIA/H3F3A**
IL-4	21	18	18
IL-10	1	0.8	0.8
IL-12B	19	16	16
INFγ	14	12	12
TNFα	3	3	3

B

Expression ratios

**gene name**	**geNorm**		**NormFinder**
	**ACTB/YWHAZ**	**ACTB/YWHAZ/ H3F3A**	**GAPDH/H3F3A**
IL-4	157	193	115
IL-10	0.8	1	0.6
IL-12B	30	37	22
INFγ	289	355	212
TNFα	5	6	3.8

C

Expression ratios

**gene name**	**geNorm**		**NormFinder**
	**ACTB/YWHAZ**	**ACTB/YWHAZ/ PPIA**	**PPIA/H3F3A**
IL-4	34	25	23
IL-10	12	9	8
IL-12B	4	3	3
INFγ	22	17	15
TNFα	6	4	4
